# EXPLORING THE EFFECTS OF RURAL AND URBAN LIVING ON LONELINESS AND REINSTITUTIONALIZATION

**DOI:** 10.1093/geroni/igz038.1961

**Published:** 2019-11-08

**Authors:** Kaleigh Ligus, Alexandra Grimaldi, Julie Robison

**Affiliations:** 1 University of Connecticut, Storrs, Connecticut, United States; 2 Yale University, New Haven, Connecticut, United States; 3 University of Connecticut, Farmington, Connecticut, United States

## Abstract

The following study employs secondary data from the Money Follows the Person Rebalancing Demonstration (MFP) in Connecticut (CT) to assess relationships between rural and urban living on loneliness and reinstitutionalization among an older adult (65+) sample. MFP is a federal initiative to help states transition people from institutional settings to the community. Older adults (n=1,301) who transitioned from institutional care to the community between 2009 and 2015 were surveyed 6, 12 and 24 months after transition. Rurality was determined according to the CT State Office of Rural Health and US Census Bureau definitions: urban area (UA), urban cluster (UC) and rural, utilizing 2017 CT Population data. SPSS was used to conduct chi-square tests and one-way ANOVAs to examine relationships. Almost half of participants (48%) resided in UAs, another 43% lived in UCs and 8% lived in rural towns. A statistically significant relationship was found between rural and UC groups and loneliness, indicated by a three-item modified version of the R-UCLA loneliness scale. Rural residents reported lower rates of loneliness (3.84 out of 9) than did UC (4.61) or UA (4.64) residents. However, a significantly higher percentage of rural residents (44%) reported at least one instance of reinstitutionalization at 24 months compared to UC (36%) or UA (30%) residents. Multivariate analyses seek to clarify these contradictory results. The findings of this study have the potential to further inform the literature regarding loneliness and connections between reinstitutionalization among older adults living in rural and urban environments.

